# Supplementary material for: Tumor microenvironment predicts local tumor extensiveness in PD-L1 positive nasopharyngeal cancer
Source: PLoS One. 2020 Mar 19;15(3):e0230449. doi: 10.1371/journal.pone.0230449 (PMC7082005; doi:10.1371/journal.pone.0230449)
Supplement: Attachment — Submitted filename: Response to Reviewers.docx [file pone.0230449.s002.docx]

Response to Reviewers:

Reviewer #1:

In this manuscript, the authors found that the PD-L1 was highly expressed in 25 NPC samples. They also revealed that denser local tumor infiltrating immune cells population have relatively much small local tumor volume. Thus, tumor infiltrating immune cells play an important role in tumor progression. The detection of tumor infiltrating immune cells may offer valuable prognostic information for NPC. This manuscript is well-written and the figures are well-presented. I only have one comment. I wonder if these NPC patents are receiving chemotherapy during this project. Chemotherapy or other therapies may have an impact on the distribution and density of tumor infiltrating immune cells. The authors should clarify this in the manuscript.

Thank you for the comment. The answer was no. All subjects included in this study did not have any prior cancer treatment. It’s true that some systemic treatments might impact tumor microenvironment. Thank you for pointing it out for us, now in our revised manuscript we have added a statement clarifying that issue.

Reviewer #2:

1.Local tumor volume can be measured by CT or MRI scan exactly. There is no need to "predict" it. In this research the authors indicated the relationship between tumor volume and its microenviroment, changing the title into, for example, "Tumor microenvironment charactaristics in different tumor extensiveness in PD-L1 positive nasopharyngeal cancer" would more appropriate.

Thank you for the comment and suggestion. Yes it is true that we can obtain exact tumor volume by CR or MRI scan. Your suggestion “Tumor microenvironment characteristics in different tumor extensiveness in PD-L1 positive nasopharyngeal cancer" was great, however, the term “in different tumor extensiveness” could be connoted by some people as different disease stages. While actually in our study we used exact tumor volume.

We used “predict” because we wanted to give the readers an upfront impression that tumor microenvironment has prognostic role when just glancing at our title. We refereed “predict” here as predicting role of tumor microenvironment. Therefore, we actually tend to stick to our original title. But we are still open to discussion if you have any other comments or ideas.

2.Did the enrolled patients received any treatment before biopsy? Medical treatment can influence the tumor progression and its microenviroment and further affect the final results.

No, all patients did not receive any prior treatment. We have revised and included a clear statement in our revised manuscript that all subjects enrolled in our study did not ever received any kind of treatment before.

3.In line 153
Did the enrolled patients have any hematological disease or infectious complications when taking peripheral blood count? These factors can affect the peripheral blood components and authors should illuminate this.

No, all patients enrolled did not have any hematological disease nor infections. The routine peripheral blood checks was part of our institution protocol. All patients would have the blood checked before biopsy and every week during treatment. Furthermore, the patient would not be biopsied if they had any kind of infection. We have also included a statement in our revised manuscript stating that all patients enrolled did not have any other severe diseases such hematological diseases or infectious diseases.

4.In line 174

It may be insufficient to use HE dye to evaluate tumor infiltrating immune cells level because it is too subjective. CD45 is widely expressed in all kinds of immune cells, so I suggest that CD45 staining is need to help evaluating the infiltrating level.

Yes, we were aware about the subjectivity. Therefore, we have actually made a precautionary step by having 2 independent pathologists blindly reviewing the HE slides. Nevertheless, we are very happy with your suggestion. Next time we will add CD45 staining to help us tackle this subjectivity issue.

5.In line 187

"imaging selected" means "selected imaging" ?

Yes, we meant selected imaging. We have revised it in our revised manuscript. Thanks for point it out for us.

6.PD-L1 is an important immunosupression molecular. So what is the relationship between PD-L1 expression and tumor infiltrating immune cells? In other kinds of cancers, PD-L1 often has higher expression in "hot tumor".

There was no relationship found between PD-L1 expression and tumor infiltrating immune cells in our study. We were aware that some tumor did have relationship exist between those two. We have added a statement in our revised manuscript describing the absence of relationship between PD-L1 and tumor infiltrating immune cells in our NPC samples.
